# Complete Sequence of Probiotic Symbioflor 2 Escherichia coli Strain G3/10 and Draft Sequences of Symbioflor 2 E. coli Strains G1/2, G4/9, G5, G6/7, and G8

**DOI:** 10.1128/genomeA.01330-14

**Published:** 2015-03-05

**Authors:** Anke Zschüttig, Christian Auerbach, Simone Meltke, Christin Eichhorn, Manuela Brandt, Jochen Blom, Alexander Goesmann, Michael Jarek, Maren Scharfe, Kurt Zimmermann, Trudy M. Wassenaar, Florian Gunzer

**Affiliations:** a Institute of Medical Microbiology and Hygiene, TU Dresden, Dresden, Germany; b Bioinformatics and Systems Biology, Justus Liebig University Giessen, Giessen, Germany; c Genome Analytics, Helmholtz Centre for Infection Research, Braunschweig, Germany; d SymbioPharm GmbH, Herborn-Hörbach, Germany; e Molecular Microbiology and Genomics Consultants, Zotzenheim, Germany

## Abstract

The complete genome of probiotic Escherichia coli strain G3/10 is presented here. In addition, the probiotic E. coli strains G1/2, G4/9, G5, G6/7, and G8 are presented in draft form. These six strains together comprise the probiotic product Symbioflor 2 (DSM 17252).

## GENOME ANNOUNCEMENT

Although probiotic activity is more often associated with lactic acid bacteria, some Escherichia coli strains have also been shown to have beneficial effects on human health. We consider it an essential step in the safety evaluation of probiotics to analyze their complete genomic content. The six probiotic E. coli strains presented here are all components of the probiotic product Symbioflor 2 (DSM 17252), provided by SymbioPharm (Herborn, Germany). The product is recommended for the treatment of irritable bowel syndrome (IBS) in adults and children (1, 2). The E. coli strains comprising Symbioflor 2 were originally isolated from a single healthy human donor and stored separately since their isolation in 1956. Based on decades of experience of clinical use in humans, and in the absence of severe adverse reactions, Symbioflor 2 can be considered safe. In February 2011, all six Symbioflor 2 E. coli strains were assigned to risk group 1 by the Central Committee on Biological Safety (ZKBS) of the German Federal Office of Consumer Protection and Food Safety (BVL).

The genome of E. coli G3/10 was sequenced at CeBiTec (University of Bielefeld, Bielefeld, Germany) using the Roche 454 Genome Sequencer FLX system (Roche Diagnostics, Mannheim, Germany). Its chromosome was initially assembled into ~190 contigs, flanked by repeated *rrn* loci, which were assembled into four large scaffolds, using the GS *de novo* assembler. Eight contigs with a total size of about 64 kb could not be assembled into any of these scaffolds. Gap closure on the scaffolds was performed by the sequencing of PCR products amplified with terminal primers and fosmid walking, resulting in four contiguous finished sequences. The three gaps between these sequences could not be closed by PCR. The strain contains one 50-kb plasmid and five smaller plasmids (Table 1). The other Symbioflor 2 E. coli strains, G1/2, G4/9, G5, G6/7, and G8, were sequenced at the Helmholtz Centre for Infection Research (Braunschweig, Germany) using the Genome Analyzer IIx (Illumina, San Diego, CA, USA). These strains contain between 1 and 5 plasmids (Table 1). *De novo* assembly was performed with Velvet (3). Gap closure was achieved by sequencing the PCR products generated with terminal primers.

**TABLE 1:** Characteristics of the six genomes

E. coli strain (DSMZ no.)^*a*^	Chromosome size (bp)	No. of contigs	Accession no.^*b*^	Plasmid(s) (size [bp])	Accession no.
G1/2 (DSM 16441)	5,090,326	252	JPKH00000000	pSYM10 (1,549), pSYM12 (7,111)	KM107846 (pSYM10), KM107848 (pSYM12)
G3/10 (DSM 16443)	4,999,267	12	JPKI00000000	pSYM1 (50,572), pSYM2 (4,197), pSYM3 (1,934), pSYM4 (1,304), pSYM5 (6,567), pSYM6 (10,433)	JN887338 (pSYM1), KM107838 (pSYM2), KM107839 (pSYM3), KM107840 (pSYM4), KM107841 (pSYM5), KM107842 (pSYM6)
G4/9 (DSM 16444)	4,545,818	79	JPKJ00000000	pSYM4	
G5 (DSM 16445)	4,787,583	151	JPKK00000000	pSYM3, pSYM7 (4,452), pSYM8 (2,353), pSYM9 (12,686), pSYM11 (3,215)	KM107843 (pSYM7), KM107844 (pSYM8), KM107845 (pSYM9), KM107847 (pSYM11)
G6/7 (DSM 16446)	5,236,262	185	JPMZ00000000	pSYM10, pSYM12	
G8 (DSM 16448)	5,160,208	300	JPKL00000000	pSYM10, pSYM12	

a Strain number in the Leibniz Institute DSMZ-German Collection of Microorganisms and Cell Cultures, Braunschweig, Germany.

b The contigs are merged into a single accession number.

The annotation of open reading frames (identified in the genomes by various automated protocols) was performed using the GenDB 2.4 annotation pipeline (4). tRNA genes were predicted using tRNAscan (5). The automated annotation was revised by BLAST analysis (6) using publicly available databases. The Symbioflor 2 strains G6/7 and G8 are closely related and may have originated from the same clone, as few differences were detected between the two draft genome sequences.

Of note is the production of microcin S by E. coli G3/10, a microcin encoded by a plasmid-carried operon (7). Microcins are peptides that function as bacteriocins against closely related species (8), and microcin S of strain G3/10 may play a role in its probiotic activity.

### Nucleotide sequence accession numbers.

This whole-genome shotgun project has been deposited at DDBJ/EMBL/GenBank under the accession numbers given in Table 1. The version described in this paper is the first version.
